# A novel probabilistic generator for large-scale gene association networks

**DOI:** 10.1371/journal.pone.0259193

**Published:** 2021-11-12

**Authors:** Tyler Grimes, Somnath Datta

**Affiliations:** Department of Biostatistics, University of Florida, Gainesville, Florida, United States of America; Instituto Nacional de Medicina Genomica, MEXICO

## Abstract

**Motivation:**

Gene expression data provide an opportunity for reverse-engineering gene-gene associations using network inference methods. However, it is difficult to assess the performance of these methods because the true underlying network is unknown in real data. Current benchmarks address this problem by subsampling a known regulatory network to conduct simulations. But the topology of regulatory networks can vary greatly across organisms or tissues, and reference-based generators—such as GeneNetWeaver—are not designed to capture this heterogeneity. This means, for example, benchmark results from the *E. coli* regulatory network will not carry over to other organisms or tissues. In contrast, probabilistic generators do not require a reference network, and they have the potential to capture a rich distribution of topologies. This makes probabilistic generators an ideal approach for obtaining a robust benchmarking of network inference methods.

**Results:**

We propose a novel probabilistic network generator that (1) provides an alternative to address the inherent limitation of reference-based generators and (2) is able to create realistic gene association networks, and (3) captures the heterogeneity found across gold-standard networks better than existing generators used in practice. Eight organism-specific and 12 human tissue-specific gold-standard association networks are considered. Several measures of global topology are used to determine the similarity of generated networks to the gold-standards. Along with demonstrating the variability of network structure across organisms and tissues, we show that the commonly used “scale-free” model is insufficient for replicating these structures.

**Availability:**

This generator is implemented in the R package “SeqNet” and is available on CRAN (https://cran.r-project.org/web/packages/SeqNet/index.html).

## Introduction

Gene expression data provide a measure of gene activity within the cells of a tissue sample. The expression of a gene is a result of a complex regulatory process that is controlled, in part, by the expression of other genes. Using the guilt-by-association principle, the co-expression of genes can be used to infer the underlying network of gene interactions [1, 2]. In this way, gene expression data enables the identification of potential regulatory interactions based on observed gene-gene associations.

There are many methods available for inferring gene regulatory networks. The methods of interest here are those that take in steady-state gene expression data to infer association networks. Steady-state data arise from non-intervention experiments that generate observational data. These experiments provide a snapshot of the underlying regulatory system.

Inferences about the association network from these data carry a lot of weight: they are used for hypothesis generation, and their results are often used to design more expensive intervention experiments [3]. Because of their key role in the guidance of early research, it is important that we utilize network inference methods that have good performance. In real datasets, the true underlying association network is unknown, so we must rely on simulation studies to benchmark network inference methods.

Simulation studies consist of four key steps: (1) generate an association network, (2) simulate gene expression data based on the network, (3) apply the network inference method to the data, and (4) compare the inferred network to the true network and assess performance. The focus of this manuscript is on the first step—generating association networks. Once a network is generated, gene expression data can be simulated as described in [5], for instance. We focus on the first step because the performance of network inference methods is heavily influenced by the topology of the underlying network [4, 5], hence it is crucial for simulated networks to resemble real association networks.

There are two approaches for generating networks: using a reference-based generator or a probabilistic generator. Reference-based methods were developed to use a gold-standard regulatory or association network from a well-studied organism like *E. coli* or yeast [6]. These methods generate networks with a topology similar to the reference, hence capturing the structure of that specific network. However, the network structure can vary greatly across organisms and tissue types. This means that a simulation using *E. coli* for a reference may not translate to realistic performance on human tissues. This presents an inherit limitation to simulation studies that use reference-based network generators. On the other hand, probabilistic generators do not require a reference, and they have the potential of sampling from a wide range of network topologies. However, existing probabilistic generators are unable to fully capture the structure of gene regulatory networks [7].

In this study, we assess the heterogeneity of gold-standard gene association networks across various organisms and tissue types. A novel probabilistic generator is proposed, and its performance is compared to existing models used in practice in terms of how well it is able to capture the variability of network structures found in the gold-standards. The results support the claim that association networks vary greatly across organisms and tissues, and the proposed method is shown to better capture this variability compared to the commonly used generators.

## Materials and methods

In this section, we begin with an overview of undirected graphs and Markov networks, which are used to define gene association networks, and we review previous work on existing network generators.

### Undirected graphs

There is a choice of whether to consider a directed or undirected network. True regulatory networks are directed, but there are latent (unobserved) structures that influence the regulatory network and are caused by the presence of unmeasured confounders, such as metabolites [8], epigenetic features [9], hormones [10], or extracellular signaling [11]. These factors should also be implemented in the simulation if a directed network is used, so that the dependencies in the simulated expression data reflect a latent structure and imitate what would be present in a real gene expression dataset. However, the topologies of real regulatory networks are not as well understood when including these latent variables into the network, which makes it difficult to determine whether generated directed networks have similar topology to real regulatory networks.

Undirected networks can model the dependence structure in observational gene expression datasets without the need to incorporate latent variables. The effect of unmeasured confounders are sufficiently represented by additional edges between genes that may not have a direct causal association (see, for example [12], for more on the relationship between directed and undirected graphs). This is how latent variables affect the topology of gold-standard association networks [13]. This makes it possible to compare the topology of generated undirected networks to various gold-standard networks.

The proposed generator is designed to create undirected graphs to simulate realistic dependence structures of observational gene expression data. In these association networks, genes are represented by nodes and dependencies are modeled by edges.

An undirected graph *G* = (*V*, *E*) is defined by a set of *n* nodes, *V* = {1, …, *n*} and edges *E* ⊂ {{*i*, *j*}|*i*, *j* ∈ *V*, *i* ≠ *j*}. An edge {*i*, *j*} ∈ *E* denotes that nodes *i* and *j* are connected in the graph. The edges can also be represented by an adjacency matrix, *A* ∈ {1, 0}^*n*×*n*^, where *A*_*i*,*j*_ = *A*_*j*,*i*_ = 1 if {*i*, *j*} ∈ *E* and is 0 otherwise.

#### Markov networks

An undirected graph can be interpreted as a Markov network, which connects the notion of “dependence” represented by edges to the joint probability distribution of the variables represented by nodes.

Consider a *n*-dimensional random vector *X* with joint distribution function *P*. The network structure encoded by *G* can be viewed as a set of independence assumptions for the distribution *P*, in which case *G* is referred to as a Markov network (or Markov random field), and *P* can be factorized over the maximal cliques of *G*. That is, *P*(*X* = *x*) = ∏_*C*∈*cl*(*G*)_ *ϕ*_*C*_(*X*_*C*_), where *cl*(*G*) is the set of maximal cliques in the graph *G*, *X*_*C*_ denotes the subset of random variables indexed by *C* ⊂ *V*, and *ϕ*_*C*_ is referred to as a clique potential (see [12] for more details).

In the Markov network, the dependency between two variables flows along the paths in the graph. Consider two distinct nodes *x* ∈ *V* and *y* ∈ *V*. If there is no path between *x* and *y* in the graph, then *x* and *y* are independent; and if they are connected directly by a single edge, then they are dependent. In these two cases, the dependencies hold regardless of any conditioning on other variables in the network. On the other hand, consider the case where there are paths connecting *x* and *y*, but no direct edge between them. Conditioning on the intervening nodes—blocking all paths between *x* and *y*—will result in conditional independence.

A gene association network is defined as the Markov network that represent the conditional dependencies in gene expression due to the true regulatory network.

Markov networks are a suitable model for observational data because they easily allow for the dependence structure to include non-causal direct associations–for example, due to unmeasured confounders—and allow the structure to contain cycles, which are a natural structure found in regulatory networks.

The network inference methods of observational expression data are tasked with hypothesis generation. The “connectivity” of each gene in an undirected network is used to determine its relative importance. Hypothesis generation can be driven from identifying hub nodes, gene modules [14], or differential network structures [15], as a few examples. These structural properties can be used to design perturbation experiments, the results of which can be combined with observational data to infer causal structures [16].

**Fig 1:**
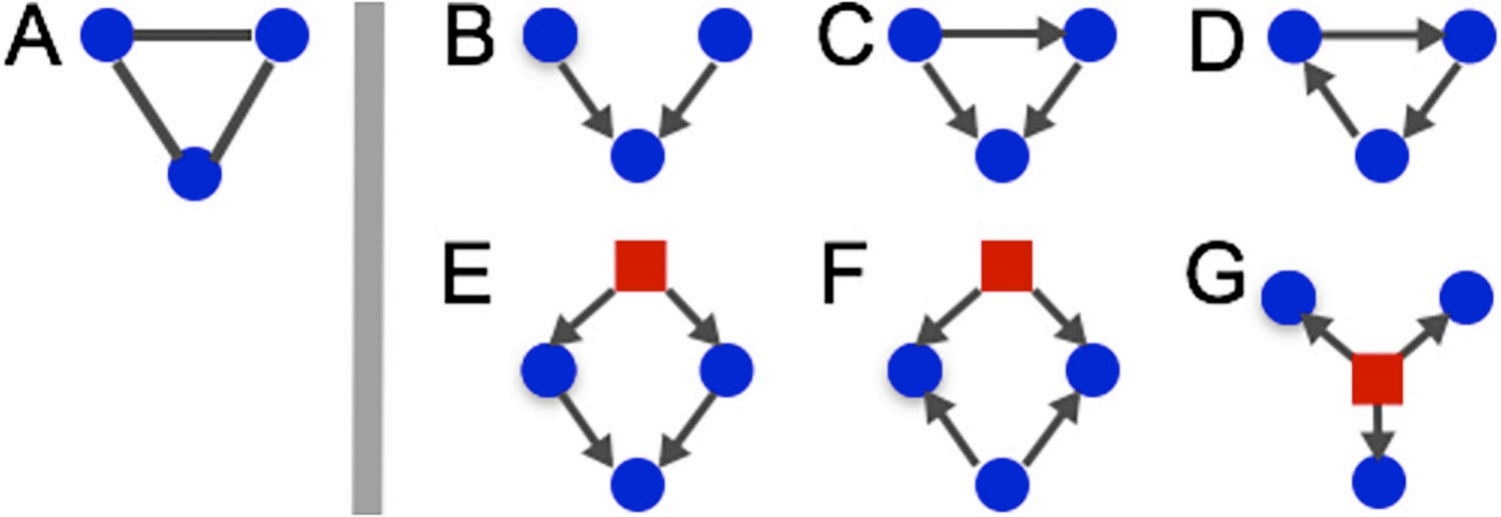
(A) The triangle structure connecting three genes in a Markov network. There are several possible directed (causal) structures that can explain these dependencies: (B) an unshielded collider, (C) a feed-forward loop, (D) a feedback loop, (E) cofactors that are both regulated by a common unmeasured variable, (F) a bi-fan in which one regulating factor is unmeasured, and (G) an unmeasured factor that regulates all three genes.

A common motif in biological networks is a triangle structure—the pair-wise association among a set of three genes [17, 18]. In an undirected network, the prevalence of this motif is characterized by a high clustering coefficient (a topological measure defined later) compared to random networks [19]. This dependence structure can arise from many different causal relationships, as illustrated in Fig 1. For example, the “v” shape in B shows a collider: two parent nodes have a direct effect on a common child node, and the parent nodes have no direct causal link. In this causal structure, the two parents have no direct connection but become conditionally dependent given information on the child node [20]. The Markov network is not able to represent the marginal independence of the v-structure, and it connects the two parent nodes due to their conditional dependence. Nonetheless, the goal of many observational studies is not to determine the precise causal structure of these motifs, but rather to identify genes that may be involved in these processes.

#### Gaussian graphical models

The Gaussian graphical model (GGM) is often used for simulating gene expression data. The GGM is an instance of a Markov network when the joint distribution *P* is modeled by a multivariate normal (Gaussian) distribution, *P* = *N*(*μ*, Σ), parameterized by a mean vector *μ* ∈ **R**^*n*^ and covariance matrix Σ ∈ **R**^*n*×*n*^. In this model, the conditional dependence structure is encoded directly in the inverse covariance matrix; variables *v*_*i*_ and *v*_*j*_ are conditionally independent given all other variables if and only if $\Sigma_{ij}^{-1}=0$ [21]. That is, if *G* is used to represent *P*, then *A*_*ij*_ = 0 if and only if $\Sigma_{ij}^{-1}=0$. This provides a useful model for generating data from an undirected graph, and it can be used to simulate gene expression data that resemble RNA-seq datasets [5].

The GGM is often used for simulation studies in current methodological research. For example [22–25], all use this approach, and they generate the gene association networks based on Erdös-Rényi [26] or Barabási-Albert [27] probabilistic generators. However, these generators do not fully capture the global structure of gold-standard association networks. And, as shown in previous studies, this underlying structure can have a substantial effect on the relative performance of network inference methods [5]. As a consequence, the relative performance in these simulation studies may not generalize to real datasets.

The proposed generator in this paper is aimed at correcting this prevalent issue. In particular, it serves to replace the probabilistic generators commonly used in simulation studies. The generated association networks can be used in a GGM or other Markov model to simulate gene expression data [5]. Performance estimates from these simulations will more closely reflect the performance on real observational gene expression data. Probabilistic generators are relied on when a reference network is unavailable—hence reference-based generators like GeneNetWeaver [28] cannot be used—and this is the setting that we are focused on.

### Previous work

This section provides a summary of existing methods for generating random networks. These methods can be divided into two approaches: reference-based generators and probabilistic generators. The former requires a reference network as input to the generator, while the latter is based on a probabilistic procedure for sampling graph structures. Reference-based generators have been useful for simulating biological networks because existing probabilistic generators are unable to capture their complex topology. The proposed methodology fills this void; it is a probabilistic generator—hence not requiring a reference network—that creates graphs with a wide range of topologies that capture the heterogeneity found in gold-standard association networks.

#### Reference-based generators

Simulation studies are conducted to benchmark the performance of network inference methods. However, the performance of any given method is usually dependent on the topology of the true network [4]. If simulated networks do not have a topological structure that resembles the true association network, then the estimated performance may not carry over to real datasets.

One solution to this problem is to use a well-studied transcription network as a reference and subsample it to simulate smaller networks. This is the approach proposed by SynTReN [29], an early simulator for gene regulatory networks. GeneNetWeaver [28] refined this approach by emphasizing module extraction rather than sampling random subnetworks from the reference [7]. By focusing on modules, GeneNetWeaver is able to preserve the topological properties of the reference network, and it was the simulator chosen for the DREAM challenge [28, 30].

The DREAM challenge used networks from both *E. coli* and yeast to establish how robust methods are to the underlying topology [30]. The benchmark results confirmed that performance varied across organisms. When studying tissue-specific networks, there is even more heterogeneity as the underlying topology may differ across tissue types [13]. Tissue-specific networks are not as well studied as E.coli or yeast, and reference networks in these cases will come with a lot of uncertainty. Because of this, it is difficult to use reference-based simulators to ascertain the robustness of network inference methods to the underlying network topology.

The main disadvantage of this class of simulators is the fact that they require a reference network. As will be shown later, the topology of gold-standard association networks varies greatly across organisms and tissues. This means that simulation studies based on a specific reference network may not generalize. Furthermore, when analyzing gene expression data from pathological networks, such as the perturbed regulatory network that may be found in cancerous cells, we cannot assume that the network topology will be similar to any given reference.

#### Probabilistic generators

Probabilistic generators do not require a reference network and instead use probabilistic models. A review of existing generators is given by [31] and is summarized here. For simple generative algorithms, the statistical properties of the network topology may be derived analytically. For more complex algorithms, however, these properties are studied using Monte Carlo experiments. Through these evaluations we can determine how well generated networks resemble gold-standard association networks.

A review of recent methodological papers showed that the Erdös-Rényi [22–24, 32–36], Watts-Strogatz [15, 33, 35, 37, 38], and Barabási-Albert [23–25, 32, 33, 35, 37, 39–46] models were most commonly used to generate networks, so these will form the basis of comparison for the proposed generator. Block diagonal network structures were also found in some papers [47–49], but these are used for specific comparisons rather than assessing general performance.

The Erdös-Rényi (ER) model [26] is one of the earliest methods developed for generating random graphs. The ER generator for a network of size *n* has a single parameter, *π*. A graph is generated in the ER model by connecting the *n* nodes at random, with each edge having probability *π* of being included. The graphs generated from this algorithm are often referred to as “random networks” due to the simplicity of the generating algorithm. For a fixed network size *n*, the ER model can access a range of distribution by varying the edge probability, *π*, but this distribution is relatively restricted and does not capture the topology of large-scale biological networks.

The Watts-Strogatz (WS) model [19] generate “small-world” networks, which resemble the topology found in many real-world networks. The WS generator has two parameters, *k* and *π*. The model initializes a graph as a ring lattice with neighborhood size *k*, and then iteratively rewires each edge with probability *π*. This algorithm leads to networks with a higher clustering coefficient and lower average path length compared to the ER model (these two metrics are discussed in Section 2.3). However, the degree distribution of these networks do not match transcription networks—in particular, the presence of high-degree hub nodes is missing.

The Barabási-Albert (BA) model [27] produces “scale-free” networks, which have a power-law degree distribution and contains hub nodes. The BA generator has three parameters, *m*_0_, *m*, and *α*. It begins with a network of *m*_0_ nodes and iteratively adds the remaining *n* − *m*_0_ nodes one at a time. Each time a node is added, it is connected to *m* of the existing nodes using a preferential attachment strategy: the *m* nodes are sampled with probability proportional to their current degree raised to the power *α*. This combined strategy of preferential attachment and iterative network growth leads to a power-law degree distribution.

The majority of the methodological papers we surveyed used the BA model to generate gene association networks for their simulation studies. However, the utility of using “scale-free” as a defining characteristic of network topology has been called into questioned [50]; there is a great variety of topologies within the class of scale-free networks, and not all scale-free networks arise through the BA model. This suggests a limitation of the BA model, but no other scale-free network generators were found in our survey.

### Problem formulation

Let **G**(*n*, *s*, *θ*) denote a probabilistic generator for networks of size *n* with a sparsity *s* parameterized by *θ*. The variables *n* and *s* are considered as design parameters, whereas *θ* are tuning parameters that modify the connective structure. Various measures of a network can be used to characterize its global topology. Let *m*(*G*) ∈ *R*^*q*^ denote a vector of such measures. A network generator is compared to a set of gold-standard datasets, $G=\{G_{1}^{*},G_{2}^{*},\ldots ,G_{k}^{*}\}$, by determining how well the generated networks *G* ∼ **G**(*n*, *s*, *θ*) capture the range of topologies found in G with respect to *m*.

In the following sections, we describe the proposed algorithm for generating networks, define nine measures used for characterizing network topology, and review the gold-standard datasets chosen for comparison. We reiterate that no comparison is made to reference-based generators like GeneNetWeaver, because those methods are not applicable in settings where no reference network is available or when the goal is to benchmark the general performance of a network inference method. The propsed method is not designed to compete with reference-based approaches, rather it is intended to be used in place of existing probabilistic generators for more general simulations of gene association networks.

### Proposed generator

The proposed network generator adopts ideas from both the WS and BA methods. However, there are two key aspects that set it apart: (1) the network is constructed by iteratively creating overlapping modular structures, and (2) it uses a more flexible model for preferential attachment. A penultimate version of this algorithm was proposed in [5]. This manuscript serves as a refinement of that algorithm and analyzes its statistical properties. The algorithm is summarized here.

**Algorithm 1**: **Generating a random network**

**Input**: *n*, the number of nodes in the graph.

**Output**: A random undirected graph.

1. Generate a random module size, *m* ∼ NB, from a negative binomial distribution.

2. Sample a subset of genes for inclusion in module. In the first module, nodes are sampled with equal probability from *V*. In all additional modules, nodes are sampled with probability that is a function of node degree. This module also includes a “link” gene, which is sampled from existing modules. This link gene acts to link modules together, forming a hierarchical structure in the graph.

3. Generate a local undirected graph for the module. This local graph is initialized as a ring lattice, similar to the WS method. It then enters a rewiring stage that takes into account the global degree of each node. After rewiring, an edge removal step is applied to control the sparsity. Finally, any disconnected components in the graph are connected to ensure that the module is a single connected component.

4. Repeat steps 1–3 until all nodes have been sampled for a module.

**Fig 2:**
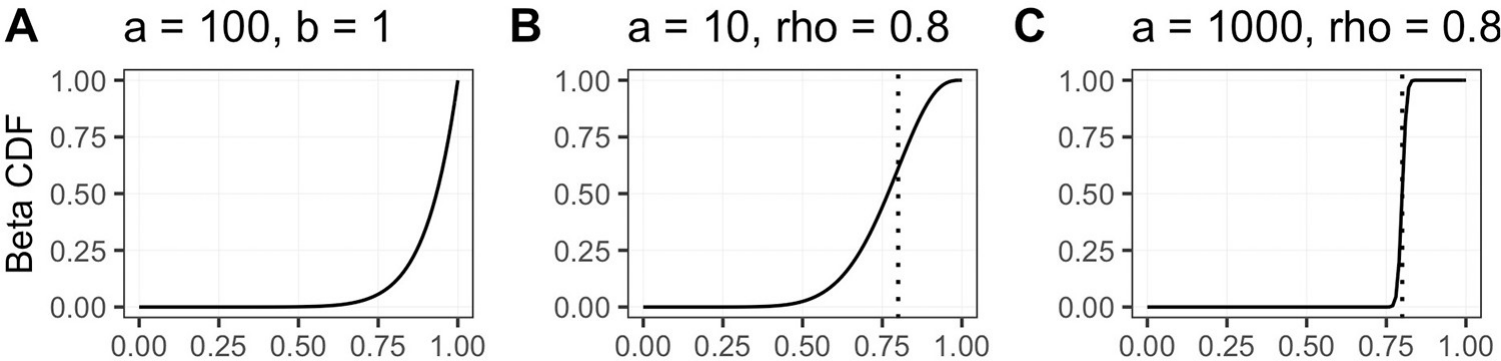
(A) The Beta distribution function with *α* = 100 and *β* = 1, (B) using the parameterization *ρ* = 0.8 with *α* = 10 we compute *β* = (10 − 1)(1 − 0.9)/0.9 − 1 = 2; the curve shows that the inflection point is at *x* = 0.8, (C) increasing *α* while keeping *ρ* constant has the effect of flattening the plateau; with *α* = 1000, we compute *β* = (1000 − 1)(1 − 0.9)/0.9 − 1 = 112, and the inflection points stays at *x* = *ρ* = 0.8. These curves illustrate the flexibility of using degree ranks along with the Beta distribution to control the preferential selection of high-degree nodes.

The preferential attachment model used by BA is proportional to the node degree, possibly raised to some power: $\pi_{i}\propto d_{i}^{\alpha }$. Two changes are made to this model in the proposed method. First, instead of using node degree, *d*_*i*_, the preferential attachment is based on the percentile, *p*_*i*_ = ∑_*j*≠*i*_ *I*(*d*_*j*_ ≤ *d*_*i*_)/*n*, of the global degree *d*_*i*_ with respect to all other nodes *j* ≠ *i* that can be sampled. Second, instead of using weights proportional to *p*_*i*_ (or to some power of it, $p_{i}^{\alpha }$), we use the Beta distribution function, *F*_*α*,*β*_(*p*_*i*_) [51]. This provides tremendous flexibility in the preferential attachment, as illustrated in Fig 2.

**Fig 3:**
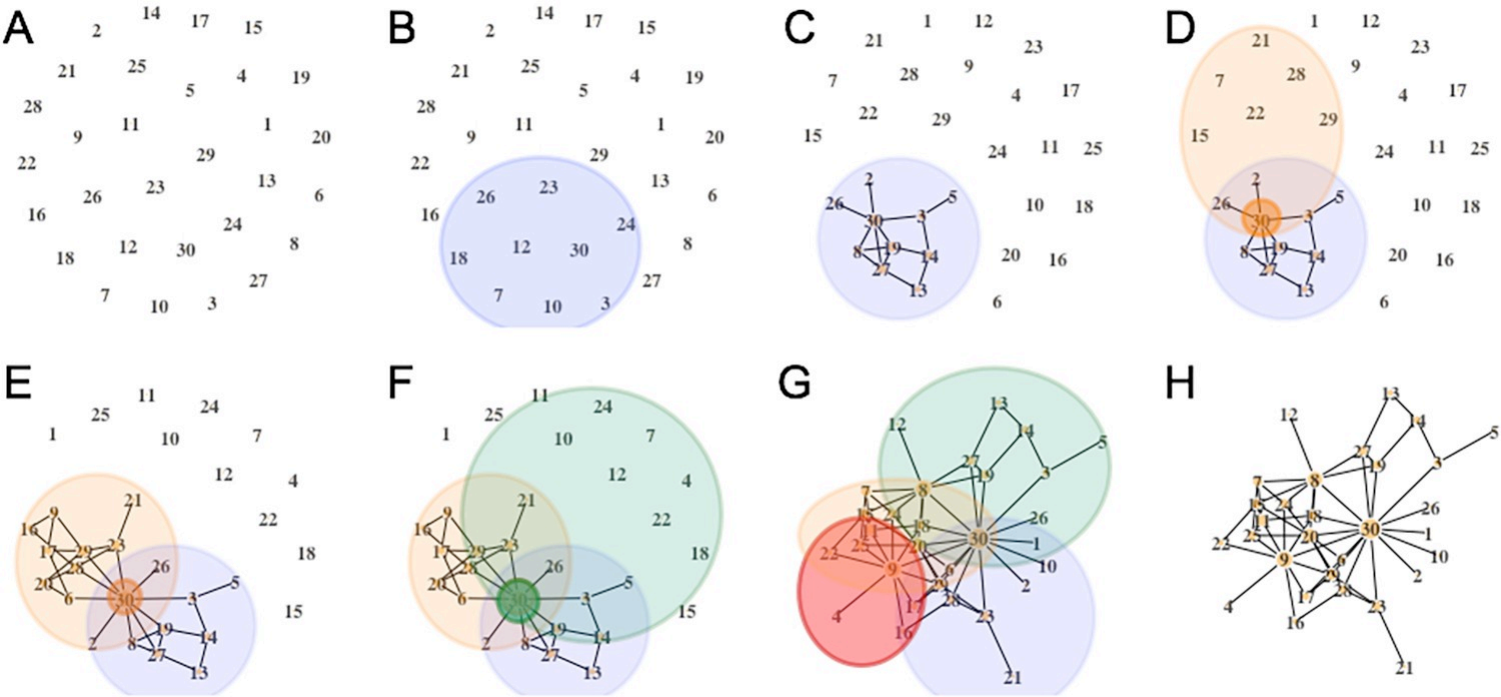
(A) The initial graph with *n* nodes, (B) random sample of nodes for the first module, (C) random network structure for the module, (D) sample a link node and populate the second module, (E) random structure for the second module, (F) continue creating modules until every node is sampled at least once, (G) the final network is composed of overlapping modules, (H) the undirected graph is the union over all modules.

An overview of the algorithm is illustrated in Fig 3. A network is generated by iteratively creating local module structures. In each iteration, the first step is to sample a “link” node from among those nodes included in existing modules (this step is skipped for the first module). This sampling uses the weights,

$$\begin{array}{c} \pi_{i}\propto F_{\alpha_{1},\beta_{1}}\left(p_{i}\right)+\epsilon , \end{array}$$

where *ϵ* is a small term added to ensure that all nodes have a nonzero chance of being selected. The tuning parameter *α*_1_ > 0 has a strong influence on the maximum degree and average path length of the global networks: if it is very large (*α*_1_ ≈ 1000), then the highest degree node is likely to be selected for the majority of modules, tightly linking all local structures together.

The next step is to finish populating the module by sampling from the set of nodes in the global network (minus the link node). This sampling uses the weights,

$$\begin{array}{c} \pi_{i}\propto \{\begin{array}{cc} \nu F_{\alpha_{2},\beta_{2}}\left(p_{i}\right), & \text{if}\;i\;\text{is}\;\text{in}\;\text{an}\;\text{existing}\;\text{module}\\ 1 & \text{if}\;i\;\text{has}\;\text{not}\;\text{been}\;\text{chosen}\;\text{for}\;\text{a}\;\text{module} \end{array} \end{array}$$

There are two important tuning parameters here (one is hidden). *ν* ∈ (0, 1) controls the degree of overlap among modules. Setting *ν* ≈ 0 will make it exceedingly unlikely for any previously chosen nodes to be picked a second time, and the majority of nodes will have minimal overlap. Increasing *ν* increases the amount of overlap, which will result in more modules being sampled to fill up the global network, hence increasing the overall number of edges and connectivity. The second parameter is hidden in *β*_2_, with *β*_2_(*ρ*) = (*α*_2_ − 1)(1 − *ρ*)/*ρ* + 1. This parameter, *ρ* ∈ (0, 1), sets the inflection point of the Beta distribution function (see Fig 2). This implementation controls the growth of nodes by placing the majority of sampling weight on the top *ρ* × 100% of highly connected genes. This allows medium- and large-degree nodes to continue to grow while preserving the low-degree nodes.

Once the module is populated with nodes, the local structure is initialized as a ring lattice, similar to the WS method, followed by a rewiring step. However unlike WS, the rewiring uses preferential attachment. A connection is rewired to a new node sampled from within the module using weights,

$$\begin{array}{c} \pi_{i}\propto F_{\alpha_{3},\beta_{3}}\left(p_{i}\right)+\epsilon , \end{array}$$

which is similar to the weighting used for sampling a link node. However, here the tuning parameters, *α*_3_ and *β*_3_, are not as essential for accessing a wide range of network topologies.

After rewiring, an edge removal step is used to randomly delete edges in the module with uniform probability; this provides a way to control the sparsity level in the network. If the edge removal step separates the module into disconnected components, then they are wired back together (using the minimum number of edges) so that the module contains a single component.

#### Design parameters

Five parameters are available in the proposed generator. Two design parameters include the network size, *n*, and the desired sparsity level, *s*, with 2/*n* ≤ *s* < 1. These two properties are often of interest when studying the performance of network inference methods, so they are meant to be specified by the user. Typically, multiple network sizes and sparsity levels will be investigated when benchmarking network inference methods. The network size, for example, will be known based on the number of genes being analyzed.

Three additional parameters, *ν*, *ρ*, and *α*_1_ enable substantial flexibility in generated topologies. In practice, the tuning parameters are randomly sampled each time the generator is run, rather than setting them by hand. This is the default approach, with $\sqrt{\nu }∼U\left(0.01,0.1\right)$, *ρ* ∼ *U*(0.5, 1), and *α*_1_ ∼ *U*(100, 1000), where *U*(*x*, *y*) denotes the uniform distribution from *x* to *y*. Note that these parameters are not meant to be inferred from the data—there is no reference network that we are trying to learn the structure of. In fact, there is no data that we’re trying to learn from at all in this context; the purpose of the simulator is to create synthetic data from scratch. These parameters should be thought of as components of the probabilistic model, and the random sampling of the parameters is a part of the stochastic process for generating a network structure.

The five design parameters are summarized below.

- *n*—The size of the network—number of nodes—to be generated.
- *s*—The desired sparsity of the network, used to set the probability of edge removal and the default neighborhood size of the initial ring lattice in each module. The sparsity will typically be a very small value, on the order of 0.01 or 0.001. Note that the sparsity of a generated network is stochastic, so the exact level may differ slightly from the set level.
- *ν* ∼ *U*(0.1, 0.1)^2^—Controls the amount of overlap among modules. Decreasing *ν* will lead to more modules being created, and hence more edges in the network. This will lead to increases in average degree, max degree, clustering coefficient, and centrality measures (with the exception of betweenness centrality), while decreasing the average path length and diameter.
- *ρ* ∼ *U*(0.5, 1)—Used to set *β* in the beta distribution *F*_*α*,*β*_ used when sampling previously selected nodes for new modules. In particular, *ρ* sets the inflection point of the distribution. Values near 1 will limit resampling to only high degree nodes.
- *α*_1_ ∼ *U*(100, 1000)—The *α* parameter in the Beta distribution used when sampling link nodes. Increasing *α*_1_ increases the maximal degree while having minimal effect on the local structure of smaller degree nodes.

### Topological measures

Nine topological measures are used to characterize the global topology: *average degree*, which is a function of the network sparsity; *max degree*; *average path length*, which considers the average shortest path (geodesic) between every pair of nodes; *diameter*, which is the length of the longest geodesic; *clustering coefficient*, which counts the number of triangle structures in the network; and four centrality measures—*betweenness*, *closeness*, *degree*, and *eigenvector centrality*—that characterize the average influence individual nodes have on the network. The definitions for each of these are provided in the Supplementary Materials. The “igraph” R package is used to compute all measures [52].

### Gold-standard datasets

The DREAM5 challenge used gold-standard networks from three different organisms and found that network inference performance was not robust across species [6]. This is a result of differences in the regulatory structures, as well as changes in prevalence of post-transcriptional modification, which reduces the correlation of expression levels between transcription factors and their targets. However, differences in network structure alone are enough to affect the relative performance of network inference methods [5].

In this study, we consider “gold-standard” association networks from eight different organisms and 12 human tissues. These networks are referred to as gold-standard, but it is important to remember that they are still prone to errors. They summarize what is currently known about the regulation mechanisms in that organism or tissue. The networks can be constructed in many different ways depending on the type and strength of evidence permitted. Different types of evidence for linking two genes include co-citation in the literature, co-expression based on high-dimensional gene expression data, co-occurance of protein domains, protein-protein interactions, or inferred co-functionality based on gene ontologies.

The purpose of these gold-standard networks in this manuscript is to illustrate the wide range of topologies that association networks may have. It is this heterogeneity that we attempt to address in the proposed network generator.

The eight organisms include *Arabidopsis thaliana* (plant) [53], *Caenorhabditis elegans* (worm) [54], *Danio rerio* (zebrafish) [55], *Drosophila melanogaster* (fly) [56], *E. coli* [57], *mus musculus* (mouse) [58], *S. cervisia* (yeast) [59], and *Zea mays* (maize) [60]. The gold-standard network for these organisms is based on positive functional gene associations. The tissue-specific human networks were obtained from HumanBase [13], and the tissues include B lymphocytes, esophagus, heart, kidney, liver, lung, mammary gland, neuron, skeletal muscle, T lymphocyte, thyroid gland, and trachea. Only edges with posterior probability greater than 0.5 were retained for the tissue-specific gold-standard networks.

### Simulation study

A simulation study is carried out to (1) evaluate the heterogeneity of topologies across the gold-standard organ and tissue-specific association networks and (2) assess the statistical properties of the probabilistic generators. The nine topological measures outlined above are computed for each reference network. The references chosen for this study cover a range of network sizes, from *n* ≈ 1000 to *n* ≈ 8000. For each network size and sparsity, 50 networks are simulated using the proposed generator. The same number of networks are also generated using the ER, WS, and BA generators defined above.

We stress again that probabilistic generators are not designed to generate networks that match the topology found in any one specific reference network. Rather, the goal is to obtain a distribution of network topologies that capture the range of network structures found across the organisms and tissues. To this end, the performance of each generator will be assessed by comparing the distribution of generated network topologies to the distribution found among the reference networks.

## Results

**Fig 4:**
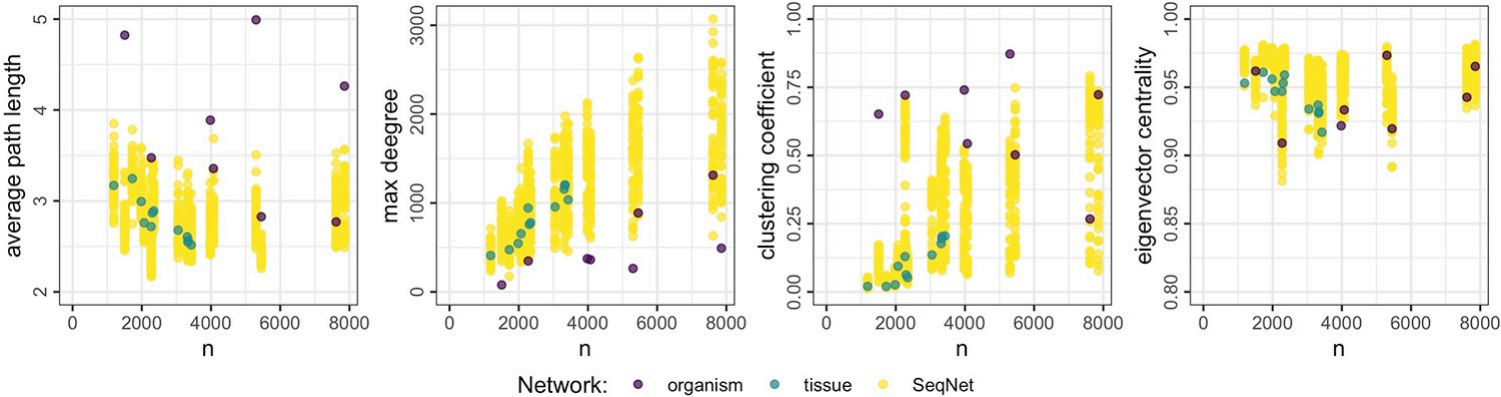
Distribution of four topological measures with respect to network size. The purple and teal dots correspond to the eight organism-specific networks and 12 human tissue-specific networks, respectively. Yellow dots show 50 simulated networks for each network size from the proposed generator. The distribution of generated topologies covers the wide variety found in the reference gold-standard networks.

**Fig 5:**
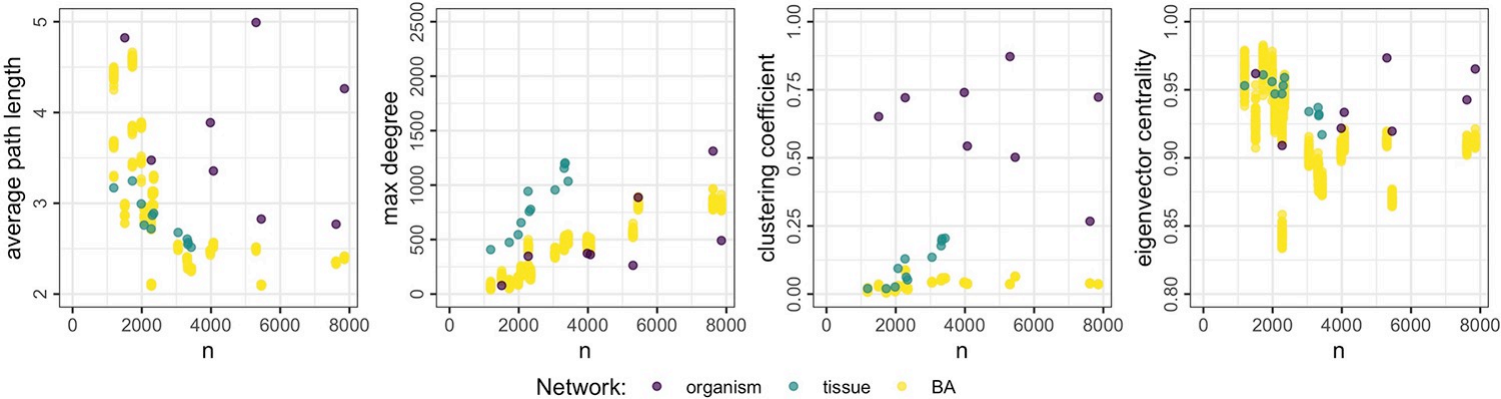
Distribution of four topological measures with respect to network size. The purple and teal dots correspond to the eight organism-specific networks and 12 human tissue-specific networks, respectively. Yellow dots show 50 simulated networks for each network size from the BA generator.

The nine topological measures are computed for the reference networks and for each generated network. Fig 4 shows the results for four of these measures (complete results are provided in the Supplementary Materials). For comparison, the same results are shown for the BA generator, which is of particular interest because this method generates “scale-free” networks and is by far the most commonly used to simulate association networks; these results are shown in Fig 5. Results for the ER and WS models are provided in the Supplementary Materials.

The first thing note from Fig 4 is the heterogeneity observed among gold-standard networks: the average path length ranges from around 2.5 to 5 across all network sizes; the max degree appears to scale linearly with network size for the tissue-specific networks, while the organism-specific networks contain hub nodes with relatively fewer connections; the clustering coefficient shows the most variation when comparing organism- to tissue-specific networks; and the eigenvector centrality has relatively less variation, but its variation persists across all network sizes.

The proposed generator covers much of the variation found in the reference networks. The average path length of simulated networks ranges from 2 to 4, similar to the reference networks, although we do find some organisms whose networks have an average path length closer to 5. The max degree scales linearly like the human tissue-specific networks, while still capturing some of the outlying organism-specific networks. The clustering coefficient and eigenvector centrality also have a wide distribution, comparable to the diversity found among the real networks.

The BA method is found to be much more restricted. For example, while it mimics the average path length of smaller networks fairly well, the max degree for those same networks is too small. For the larger networks, the average path length is not captured well at all. It is also unable to simulate the high clustering coefficient found in the organism-specific networks; this means that those important structures described in Fig 1 will be under represented in generated networks.

**Fig 6:**
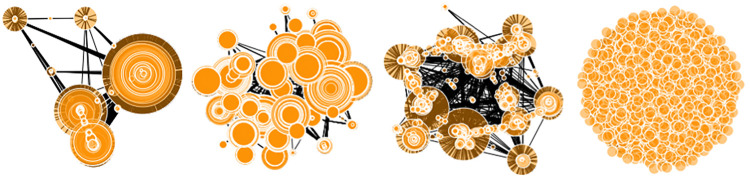
From left to right: The T lymphocyte tissue-specific network, the *C. elegans* network, a random network from the proposed generator, and a random network from the BA model.

For a visual comparison, Fig 6 shows theT lymphocyte tissue-specific network, the *C. elegans* organism network, a random network generated from the proposed generator, and a random network from the BA model. Each network contains approximately 2200 nodes and a sparsity of around 0.01.

In this visualization, the presence of large hub nodes in the two gold-standard association networks stands out, and the hierarchical structure is partially observable because the hub nodes are separated due to their low interconnectedness. The simulated network from the proposed generator is visibly similar to the two gold-standard networks in both of these regards. This is in contrast to the BA network (on the far right), whose structure is visually blocked because the minimum degree of each node is high (which is necessary to obtain the desired sparsity in this model). While the BA model does provide a network with a scale-free degree distribution, it is clear that this characterization alone is insufficient for representing gold-standard association networks.

## Discussion

Observational gene expression data are an important resource for hypothesis generation and guiding experimental research of gene regulatory networks. The relative performance of expression-based network inference methods has a strong dependence on the underlying network topology. As an example of this, we encourage the reader to view the simulation results from the penultimate version of this generator in [4], which outlines a framework for generating realistic expression data from an association network. The results of that paper highlighted the need for a comprehensive assessment of the variability in gold-standard association networks across different organisms and tissue types, along with a careful refinement of the proposed network generator to maximize its coverage of those topologies.

We find that the global topology—measured using a variety of metrics—of gold-standard association networks vary greatly across organisms and tissues. This heterogeneity limits the generalizability of simulation studies that use reference-based network generators, since those will only capture the topology of the reference provided. Similarly, the probabilistic generators that are commonly used in this context are unable to capture this rich distribution of network structures. The major consequence of these limitations is that benchmarks of network inference methods are not able to properly assess performance. This is well known and, for example, the DREAM challenge addressed this issue by evaluating methods using data from multiple species. But problem continues to grow: as we begin to collect and analyze expression data on more tissue-types and new organisms, it’s unclear how well benchmarked methods should be expected to perform on these new data.

The proposed probabilistic generator will allow simulation studies to consider a much wider range of plausible association networks compared to currently used generators. Importantly, this allows methodological researchers to explore the types of structures that an expression-based network inference method performs well on and where it performs poorly. By simulating thousands of networks from this generator, and using the framework described in [5] to simulate gene expression data from them, the inference method will be exposed to a wide range of topologies—essentially a wide range of potential organisms and tissue types—enabling a more complete evaluation of how well that method will work in general.

A reviewer pointed out that the range of topologies created by the proposed generator may be too broad and many of the networks may be unrealistic. This is a reasonable concern, and we have a few thoughts on it: (1) although gold-standard association networks are used here, we must remember that these only summarize our current knowledge and are subject to change. We suspect that the variability observed in this study will only continue to grow as more organisms and tissue types are studied. This would follow the trend of diversity in the most well-studied organisms such as *E. coli* and *S. cerevisiae* (yeast), which have substantially different networks. Hence, it’s unclear to us at this point whether or not the range captured by the proposed generator really is too broad.

(2) If the user still wants to restrict the range of topologies generated, then the generated networks can be filtered. This can be done in an automatic way by setting up a specific criterion for one or more topological measures. For example, if the clustering coefficient is expected to be between 0 and 0.25 for a realistic network, then any generated networks that fall outside of that range is discarded and resampled. This will require generating more networks overall, but the computational cost of generating a network in this algorithm is low: the algorithm runs in linear time with the number of nodes, *O*(*n*), and takes less than a second to generate a network of *n* = 1000 nodes on a standard laptop computer.

(3) Lastly, we recommend conducting simulation studies in an investigative way. Suppose that a particular network inference method is found to perform poorly in 10% of generated networks. As part of the simulation study, the topology of that 10% should be studied to see how those networks differ from the other 90%. This may reveal, for instance, that the method performs poorly when there are too many hub genes or the clustering coefficient is too high. Benchmark results that incorporate this topological information will provide a more complete picture of relative performance. The researcher referring to that benchmark can then decide which topological properties are “realistic” and choose a method accordingly.

We surveyed the literature to determine which probabilistic generators are used in practice for simulating gene association networks. By far the most frequently used is the BA model. This is because gene association networks are often characterized as having the scale-free property, which makes the BA model a natural choice. However, the results from this study show that the scale-free class of networks defined by the BA model does not adequately capture the topologies found in current gold-standard networks. This supports previous findings on the limitations of the BA model [50].

The other generators found in use are the ER and WS models. We show that these are also insufficient for simulating gene association networks. Incidentally, as a reviewer pointed out, there may be other generators that could be considered for this application, such as the stochastic block model. However, the comparison made here is restricted to the models currently used in practice. The intention of the proposed generator is to provide an alternative to the standard models used in this context, without requiring any substantial tuning or modification from the user. We suspect that one reason for the frequent use of the BA model is that it doesn’t require any model fitting. By design, it automatically generates networks with a scale-free structure. This ease-of-use is in contrast to more general models, such as the stochastic block model., which would require a model fitting step, and the user would need to obtain several reference networks to get started. The proposed model is designed specifically with gene association networks in mind so that it can be used, like the BA model, without the need for any model fitting.

We reiterate that no comparison is made to reference-based networks, such as GeneNetWeaver, because those methods are not applicable for the context we are interested in: if no reference network is available, or if the study is intended to obtain benchmarks that generalize across organisms and tissues, then the reference-based approach cannot be used and the proposed probabilistic generator is an ideal choice. However, for studies that do focus on a specific organism or tissue, and a reference network is available for that target, then reference-based generators are still recommended.

## Supporting information

S1 Text Supplementary material. The mathematical definitions for the nine topological measures are provided, along with the complete simulation results comparing the four probabilistic generators.
(PDF)
