# Supplementary material for: A novel probabilistic generator for large-scale gene association networks
Source: PLoS One. 2021 Nov 12;16(11):e0259193. doi: 10.1371/journal.pone.0259193 (PMC8589155; doi:10.1371/journal.pone.0259193)
Supplement: S1 Text — The mathematical definitions for the nine topological measures are provided, along with the complete simulation results comparing the four probabilistic generators. (PDF) [file pone.0259193.s001.pdf]

Supplementary Material for "A novel probabilistic generator for large-scale gene association networks"

Tyler Grimes and Somnath Datta

June 7, 2021

This supplement provides the mathematical definitions for the nine topological measures. The complete simulation results comparing the proposed generator and existing generators (ER, WS, and BA) to the reference datasets are also provided.

1 Topological measures

Nine topological measures were chosen to characterize the network structure. These measures provide a variety of ways to summarize the global connectivity in the network. The "igraph" R package is used to compute all measures (Csardi and Nepusz, 2006).

1. Average degree
$$\bar{d}(G) = n^{-1} \sum_{v \in G} d(v).$$
Note that sparsity is related to average degree by $s(G) = \bar{d}(G)/n$.

2. Max degree
$$d_{\max}(G) = \max_{v \in G} d(v).$$

3. Average path length
$$L(G) = \frac{1}{n(n-1)} \sum_{v \in G} \sum_{w \in G} \ell(v, w),$$
where $\ell(v, w)$ denotes the length of the shortest path (geodesic) between nodes v and w.

4. Diameter
$$D(G) = \max_{v, w \in G} \ell(v, w).$$

5. Clustering coefficient

$$Cl(G) = \frac{\#\text{ of triangles in } G}{\#\text{ of connected triples in } G}.$$

Four centrality measures are also considered. The graph-level centrality score is based on node-level centrality; it has the general form, $C(G) = \sum_{v \in G}\{\max_{w \in G} c(w) - c(v)\}$, and is normalized by c^*, the maximum value of $c(v)$ over all possible networks of the same size as G. Small values of C indicate that all nodes have the same centrality, and large values indicate that most nodes have minimal centrality.

6. Betweenness centrality

$$c_B(v) = \sum_{i \leq j} g_{ij}(v)/g_{ij} \text{ and } c_B^* = (n-1)(n-2)/2,$$

where g_{ij} is the number of geodesics connecting nodes i and j, and $g_{ij}(v)$ is the number of those geodesics containing v.

7. Closeness centrality

$$c_C(v) = [\sum_{w \in G} \ell(v,w)]^{-1} \text{ and } c_C^* = (n-1)^{-1}.$$

8. Degree centrality

$$c_D(v) = d(v) \text{ and } c_D^* = n-1.$$

9. Eigenvector centrality

$$c_E(v) = \frac{1}{\lambda} \sum_{w \neq v} I((v,w) \in E) c_E(w),$$

where $I((v,w) \in E)$ is an indicator of whether nodes v and w are connected. This is a recursive definition that measures centrality of a node based on the centrality of its neighbors. These centrality scores correspond to the values of the first eigenvector of the network's adjacency matrix (Wasserman *et al.*, 1994).

2 Additional Results

The complete simulation results are provided in this section. The results are separated by each topological measure, so that the four generators can be easily compared.

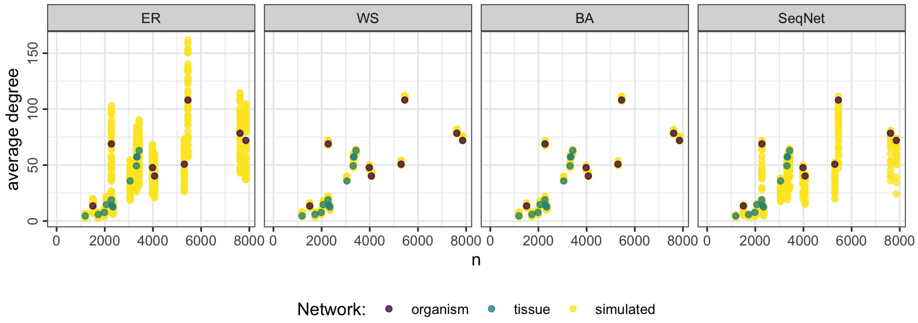

Figure 1: Distribution of the average degree with respect to network size. The purple and teal dots correspond to the eight organism-specific networks and 12 human tissue-specific networks, respectively. Yellow dots show 50 simulated networks for each network size.

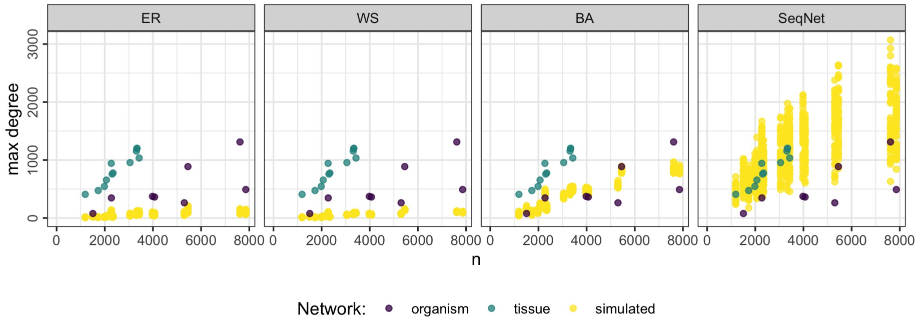

Figure 2: Distribution of the max degree with respect to network size. The purple and teal dots correspond to the eight organism-specific networks and 12 human tissue-specific networks, respectively. Yellow dots show 50 simulated networks for each network size.

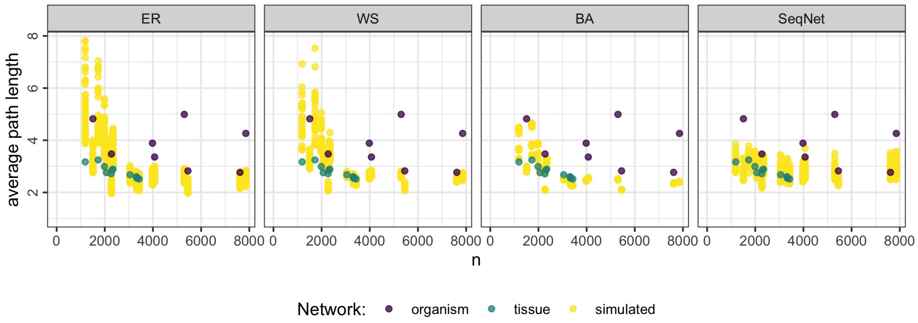

Figure 3: Distribution of the average path length with respect to network size. The purple and teal dots correspond to the eight organism-specific networks and 12 human tissue-specific networks, respectively. Yellow dots show 50 simulated networks for each network size.

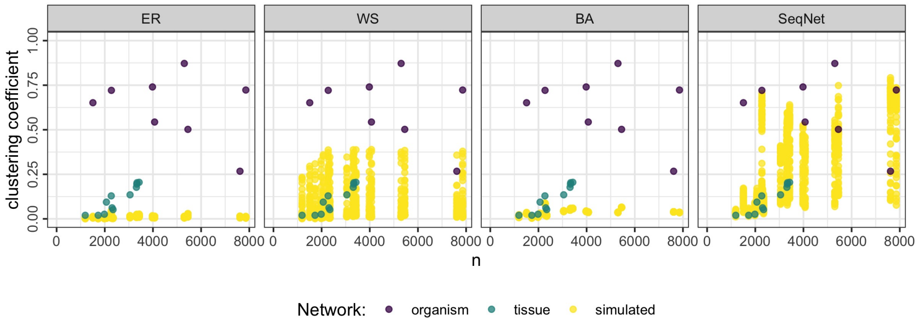

Figure 5: Distribution of the clustering coefficient with respect to network size. The purple and teal dots correspond to the eight organism-specific networks and 12 human tissue-specific networks, respectively. Yellow dots show 50 simulated networks for each network size.

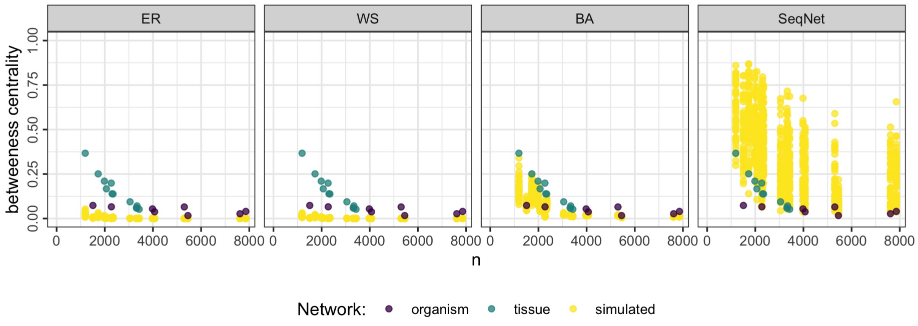

Figure 6: Distribution of the betweeness centrality with respect to network size. The purple and teal dots correspond to the eight organism-specific networks and 12 human tissue-specific networks, respectively. Yellow dots show 50 simulated networks for each network size.

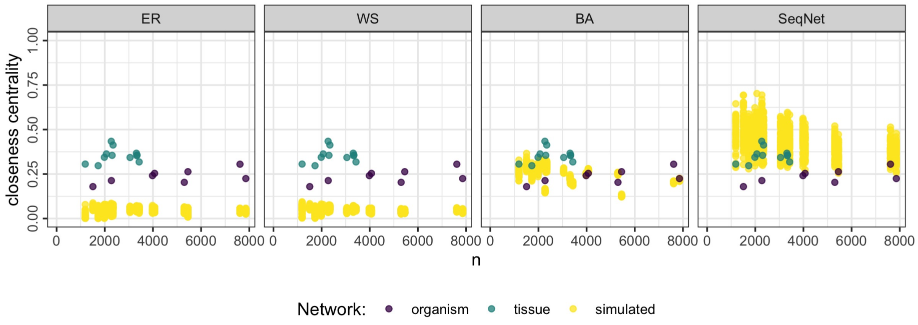

Figure 7: Distribution of the closeness centrality with respect to network size. The purple and teal dots correspond to the eight organism-specific networks and 12 human tissue-specific networks, respectively. Yellow dots show 50 simulated networks for each network size.

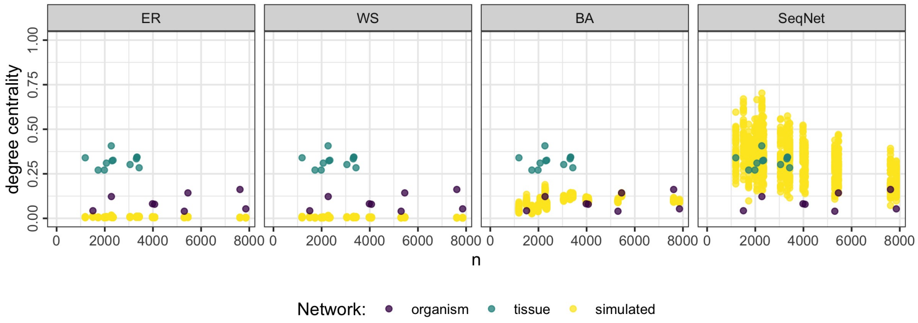

Figure 8: Distribution of the degree centrality with respect to network size. The purple and teal dots correspond to the eight organism-specific networks and 12 human tissue-specific networks, respectively. Yellow dots show 50 simulated networks for each network size.

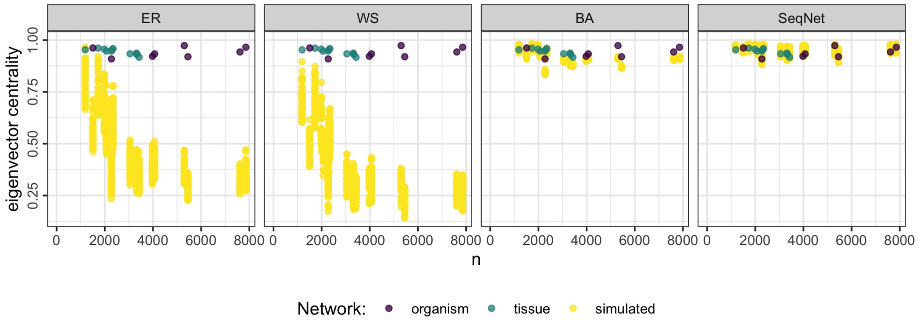

Figure 9: Distribution of the eigenvector centrality with respect to network size. The purple and teal dots correspond to the eight organism-specific networks and 12 human tissue-specific networks, respectively. Yellow dots show 50 simulated networks for each network size.

References

Csardi, G. and Nepusz, T. (2006). The igraph software package for complex network research. *InterJournal*, **Complex Systems**, 1695.

Wasserman, S., Faust, K., *et al.* (1994). *Social Network Analysis: Methods and Applications*, volume 8. Cambridge University Press.

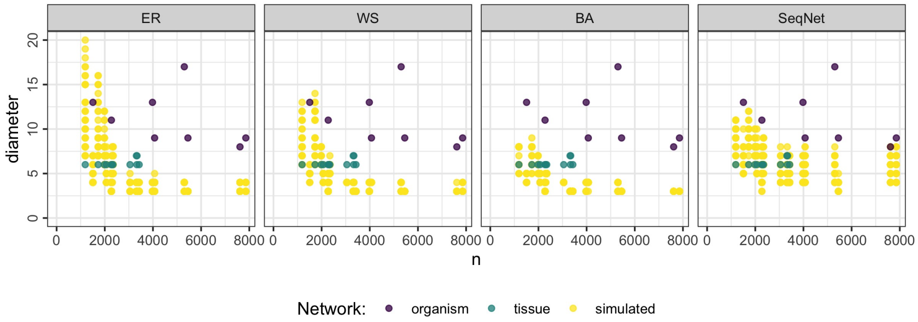

Figure 4: Distribution of the diameter with respect to network size. The purple and teal dots correspond to the eight organism-specific networks and 12 human tissue-specific networks, respectively. Yellow dots show 50 simulated networks for each network size.
